# Protection motivation theory-based management after surgery for osteoporotic vertebral fractures: Modulating inflammatory factors and pain mediators, and ameliorating hypercoagulability and bone metabolism

**DOI:** 10.1097/MD.0000000000048184

**Published:** 2026-04-24

**Authors:** Xiaorong Guo, Xuan Wei, Xiaojuan Zhang, Jing Liu

**Affiliations:** a Department of Orthopaedics, Affiliated Hospital of Gansu University of Chinese Medicine, Lanzhou, Gansu Province, China; b Department of Spine Surgery, Affiliated Hospital of Gansu University of Chinese Medicine, Lanzhou, Gansu Province, China.

**Keywords:** bone metabolism, coagulation function, inflammatory factors, osteoporotic vertebral fractures, pain mediators

## Abstract

The purpose of this research was to investigate how the protection motivation theory (PMT)-based management model influences inflammatory factors, pain mediators, coagulation function, and bone metabolism in postsurgical osteoporotic vertebral fracture (OVF) patients. Between February 2024 and March 2025, OVF patients undergoing surgery at our center were recruited for this investigation. Ultimately, 74 patients were assigned to the PMT group, compared with 71 who were designated as controls. For each participant, venous blood sampling under fasting conditions was performed preoperatively and on postoperative day 5. We then measured a comprehensive set of parameters using dedicated techniques: inflammatory factors (interleukin-1 beta [IL-1β], interleukin-6, interleukin-10 [IL-10], tumor necrosis factor-α, high-sensitivity C-reactive protein [hs-CRP]) and bone metabolism markers (C-terminal telopeptide of type I collagen [CTX], bone Gla protein [BGP], osteocalcin [OC]) via enzyme-linked immunosorbent assay, pain mediators (substance P [SP], prostaglandin E2 [PGE_2_]) via radioimmunoassay, oxidative stress markers (malondialdehyde [MDA], superoxide dismutase) via the thiobarbituric acid method, and coagulation parameters (D-dimer [D-D], fibrinogen [FIB], P-selectin, platelet) via an automatic coagulation analyzer. The resulting data were subjected to both intragroup and intergroup comparative analyses. In addition, the adverse reactions during the perioperative period were counted, and the Japanese Orthopaedic Association (JOA) scores and Barthel index (BI) questionnaires were conducted before and 1 month after the operation to evaluate the rehabilitation quality of the patients. Surgical intervention resulted in an increase in IL-1β, interleukin-6, tumor necrosis factor-α, hs-CRP, SP, PGE_2_, MDA, D-D, FIB, P-selectin, and CTX, as well as a decrease in IL-10, superoxide dismutase, BGP, and OC in both cohorts (*P* < .05). According to intergroup analyses, the PMT group exhibited lower IL-1β, hs-CRP, SP, PGE_2_, MDA, D-D, FIB, and CTX, along with higher IL-10, platelet, BGP, and OC, than controls (*P* < .05). The incidence of perioperative deep vein thrombosis in the PMT group was lower than that in the control group (*P* < .05). Although the JOA and BI of the 2 groups were higher than those before operation, the JOA and BI of the PMT group were higher than those of the control group at 1 month after operation (*P* < .05). Early postoperative adoption of PMT-based management following OVF surgery contributes to significant clinical improvements by suppressing systemic inflammation, limiting pain mediator synthesis, ameliorating hypercoagulability, and normalizing disrupted bone metabolism.

## 1. Introduction

Osteoporotic vertebral fractures (OVFs) stand out as one of the most severe consequences of osteoporosis.^[1]^ Epidemiological data reveal that among individuals aged 50 and above, fractures linked to osteoporosis affect about 33% of females and 20% of males. Notably, OVFs make up an impressive 20% to 30% of these incidents.^[2]^ Surgery remains an effective approach for pain relief and spinal stabilization. However, postsurgical patients often encounter multiple pathological challenges. On one side, persistent inflammation may arise due to surgical trauma and comorbid osteoporosis, leading to the massive release of pro-inflammatory markers.^[3]^ On the other side, nerve damage and tissue repair processes activate the secretion of pain-inducing mediators, elevating the likelihood of chronic pain development.^[4]^ Meanwhile, being immobile after surgery and having a hypercoagulable blood state are likely to cause deep vein thrombosis (DVT). In addition, osteoporosis-induced bone metabolism imbalance – marked by reduced osteoblast activity and overactive osteoclasts – can slow down fracture healing and raise the risk of re-fractures.^[5]^ These pathophysiological changes are intertwined, which have a profound impact on patients’ postoperative rehabilitation quality and their long-term prognoses.

In recent years, the protection motivation theory (PMT) has gained increasing attention as a theoretical framework for guiding health behavior interventions in rehabilitation settings.^[6]^ Its core proposition suggests that motivation to adopt protective behaviors arises from 2 appraisal pathways: threat appraisal (perceived severity and susceptibility) and coping appraisal (perceived self-efficacy and response efficacy). Together, these evaluations stimulate intentional efforts toward behavioral adaptation.^[7]^ Evidence has indicated PMT’s capacity for improving adherence to glycemic control in diabetics.^[8]^ However, its application in post-orthopedic surgery management remains exploratory, with a notable lack of studies on mechanism-based interventions for postsurgical pathophysiological markers. Besides, most existing studies focus on PMT’s impact on patients’ subjective behaviors (e.g., medication compliance and follow-up intention) while paying little attention to the dynamic regulation of objective pathological indicators like inflammatory factors, pain mediators, coagulation function, and bone metabolism. The multifactorial pathology following OVF surgery – such as the interaction between inflammation, pain, hypercoagulability, and bone metabolism imbalance – has not been thoroughly investigated. The ability of PMT to halt this interconnected pathological cascade via motivation-based strategies is therefore still unknown.

Focusing on surgically treated OVF patients, this study conducted a comprehensive analysis of how PMT modulates a range of physiological factors, including inflammatory cytokines, pain mediators, coagulation metrics, and bone turnover. Specifically, our research team studied the anti-inflammatory effect of PMT after OVF surgery, as well as its role in relieving pain, reducing blood hypercoagulability, and improving bone metabolism. To our knowledge, this is the first instance where PMT is correlated with objective pathological indices to explain its regulatory role in the multisystem pathological processes of OVF patients post-surgery, analyzed from molecular and cellular perspectives. The results support the development of an innovative framework centered on “motivation-driven engagement, pathological alleviation, and rehabilitation refinement.”

## 2. Materials and methods

### 2.1. Study subjects

This study was approved by the Ethics Committee of Affiliated Hospital of Gansu University of Chinese Medicine (Approval No.: 2025-GSUCM-0814). This prospective cohort analysis enrolled 164 individuals diagnosed with OVFs and admitted to our institution from February 2024 to March 2025. Group size was determined as 62 subjects each, based on the primary outcome measure of postoperative variation in interleukin-6 (IL-6) from baseline (assuming two-sided significance level α = 0.05, β = 0.2, effect size *d* = 0.8). To allow for a 20% dropout rate, a minimum of 69 participants per group were targeted for enrollment. This study ultimately enrolled 145 patients following screening based on the inclusion and exclusion criteria. These participants were divided into 2 groups: 74 patients constituted the PMT group and received postoperative management with PMT, and 71 comprised the control group and received conventional postoperative care. The duration of the postoperative management intervention was 5 days for both groups. All participants signed an informed consent form.

### 2.2. Inclusion and exclusion criteria

Inclusion criteria are as follows: age ≥ 50 years; an osteoporosis diagnosis,^[9]^ defined as a bone mineral density *T*-score of ≤−2.5 standard deviation (SD) at the lumbar spine or hip, as measured by dual-energy X-ray absorptiometry; presence of a imaging-confirmed fresh OVF (symptom duration ≤ 3 months, with ≥20% vertebral compression); and undergoing surgical intervention (percutaneous vertebroplasty, kyphoplasty, or open posterior pedicle screw instrumentation and fusion) performed by a consistent surgical team, with stable vital signs within 72 hours postoperation.

The following are the exclusion criteria: pathological fractures secondary to tumors, infection, or metabolic bone disease; severe concomitant cardiac, hepatic, or renal dysfunction; hematologic disorders or labile anticoagulant/antiplatelet medications; psychiatric conditions; recent (3-month) administration of drugs affecting bone turnover (bisphosphonates or denosumab); and current pregnancy or breastfeeding.

### 2.3. Sample collection

Four milliliters of fasting venous blood were drawn from the antecubital vein of each participant twice: once before the surgery (within 24 hours of admission) and once after the management intervention (on the 5th day of the intervention). Blood was drawn into both anticoagulant and coagulation-promoting tubes accordingly. Specimens in the coagulation-promoting tubes were left at room temperature for 30 minutes and then centrifuged (1505 × g, 4°C, 15 minutes) to separate the serum. The separated serum was stored at −80°C for later testing, with strict avoidance of repeated freezing and thawing. The blood samples in the anticoagulant tubes were tested within 2 hours of collection.

### 2.4. Laboratory testing

Enzyme-linked immunosorbent assay kits were employed to quantify interleukin-1 beta (IL-1β), IL-6, interleukin-10 (IL-10), tumor necrosis factor-alpha (TNF-α), prostaglandin E2 (PGE_2_), superoxide dismutase (SOD), C-terminal telopeptide of type I collagen (CTX), bone Gla protein (BGP), and osteocalcin (OC) levels: each well received standard (50 µL) or plasma (25 µL) combined with diluent (25 µL, 1% bovine serum albumin in phosphate-buffered saline). After sealing and incubating at 4°C for 1 hour (using 1% bovine serum albumin in phosphate-buffered saline as blocking buffer), 100 µL of biotin-conjugated detection antibody was introduced, followed by a 1-hour incubation at 37°C. One hundred microliters of streptavidin-horseradish peroxidase (diluted 1:200) was added and incubated at 37°C for 30 minutes. The reaction was terminated by adding 50 µL of 2N H_2_SO_4_, and absorbance (OD) at 450 nm was measured using a microplate reader. Sample concentrations were determined based on a standard curve fitted with a 4-parameter logistic model. Quality control (QC) sera (high, medium, and low concentrations) provided by the manufacturer were used, and QC was performed daily before testing, requiring the results to fall within the range of ±2SD.

The measurement of substance P (SP) was conducted via radioimmunoassay: plasma and diluted SP antiserum (1:20), each 100 μL, were incubated together for 24 hours at 4°C. One hundred microliters of ^125^I-SP tracer was introduced, and the incubation was prolonged for 24 hours. Precipitation was achieved by adding 200 μL of polyethylene glycol and centrifuging at 3000 × g for 15 minutes, followed by supernatant removal. The radioactivity (cpm) of the precipitate was measured using a γ-counter. The SP concentration was determined based on a standard curve ranging from 0.1 to 20 pg/mL. Daily quality assurance involved 2 monitoring runs of pooled serum samples incorporated with SP, requiring the coefficient of variation to be <10%.

The thiobarbituric acid method was adopted for malondialdehyde (MDA) determination: one mL of thiobarbituric acid working solution (comprising 15% trichloroacetic acid, 0.375% thiobarbituric acid, and 0.25% butylated hydroxytoluene) was mixed with plasma and placed in a boiling water bath for 15 minutes. After cooling, the mixture was centrifuged (1000 × g, 10 minutes) to collect the supernatant. Following the color development step, the reaction mixture was cooled and subsequently centrifuged at 1000 × g for 10 minutes to obtain the supernatant. The absorbance of the supernatant was measured at 532 nm with a ultraviolet-visible spectrophotometer. The MDA concentration (nmol/mL) was determined by interpolation from a standard curve generated with TEMAN (1,1,3,3-tetraethoxypropane) standards across a concentration range of 0 to 20 nmol/mL.

D-dimer (D-D), fibrinogen (FIB), P-selectin, platelet (PLT), and high-sensitivity C-reactive protein (hs-CRP) measurements were conducted using a coagulation analyzer (Sysmex CS-5100) and an automatic hematology analyzer (Mindray BC-5180). After sample loading, the instruments automatically performed the detection process and output the results. Instrument-matched QC plasmas (high, medium, and low concentrations) were analyzed each day prior to patient sample testing. Acceptance criteria for the assay required that these QC results be within ±2SD.

Laboratory personnel performing biochemical assays were blinded to group allocation to minimize measurement bias. However, because the intervention involved behavioral management based on PMT, blinding of participants and clinical care providers was not feasible.

### 2.5. Questionnaire survey

Before and 5 days after surgery, the pain of patients was evaluated by visual analogue scale (VAS)^[10]^ (1–10 points), and the higher the score, the more obvious the pain experience. In addition, the patients were followed up for 1 month, and the spinal function of the patients was evaluated by the Japanese Orthopaedic Association (JOA) score^[11]^ before and 1 month after the operation. JOA includes sensory, motor, bladder function, and other dimensions (the total score is 29, and the higher the score, the better the function). Barthel index (BI)^[12]^ was used to evaluate the living ability of patients. BI included eating, dressing, walking, and 10 other items (the full score was 100 points, ≥60 points were basic self-care). Pain intensity (VAS) was assessed preoperatively and on postoperative day 5. Functional recovery was evaluated using JOA and BI scores preoperatively and again at 1 month postoperatively during follow-up.

### 2.6. Postoperative safety assessment

Patients were monitored for adverse events and complications during the intervention, such as DVT, dizziness/headache, abdominal pain/diarrhea, and other events.

### 2.7. Quality control

Prior to the intervention, nursing and rehabilitation therapists were required to complete a uniform instruction program on PMT theory and practice, only permitting those who successfully passed the evaluation to take part. In addition, a dedicated electronic database was created. To ensure data accuracy, entries were performed independently by 2 individuals and then cross-verified. Any missing information was obtained via telephone follow-up and subsequently added. Laboratory personnel, who were blinded to the patient group assignments, were responsible for all sample collection and indicator analysis.

### 2.8. Statistical analysis

Data processing and analysis were carried out with SPSS 30.0. The chi-square test or Fisher exact test was used for comparing categorical variables (n [%]). Continuous variables were tested for normality with the Shapiro–Wilk method. Those normally distributed are presented as $\left(\bar{x}\pm s\right)$ and compared between groups with an independent *t* test; paired *t* tests were used for within-group comparisons. Non-normal variables are summarized as M (P25, P75) and compared between groups with the Mann–Whitney *U* test, or within groups via the Kruskal–Wallis test. Differences achieving a *P* value of <.05 were considered statistically significant.

## 3. Results

### 3.1. Comparability in clinical characteristics between groups

A critical step in this cohort study involved verifying the comparability of the groups to minimize confounders. An examination of clinical variables, including patient age, sex, surgical intervention, and postoperative hospital duration, showed no notable disparities (*P* > .05). This similarity lends credibility to the results observed in the study (Table 1).

**Table 1:** Clinical data of 2 groups.

Projects		Control group (n = 71)	PMT group (n = 74)	Statistics, *t* or χ^2^	*P*
Age		65.66 ± 6.03	64.46 ± 6.34	1.169	.244
Sex	Male	26 (36.62%)	31 (41.89%)	0.422	.516
	Female	45 (63.38%)	43 (58.11%)		
Type of surgery	Percutaneous vertebroplasty	34 (47.89%)	31 (41.89%)	0.528	.768
	Percutaneous kyphoplasty	30 (42.25%)	35 (47.30%)		
	Open internal fixation surgery	7 (9.86%)	8 (10.81%)		
Postoperative hospital stay (d)		7.25 ± 1.17	7.28 ± 1.03	0.166	.869
Combined with diabetes mellitus	Yes	55 (77.46%)	62 (83.78%)	0.929	.335
	No	16 (22.54%)	12 (16.22%)		
Combined hypertension	Yes	42 (59.15%)	38 (51.35%)	0.892	.345
	No	29 (40.85%)	36 (48.65%)	–	–
Duration of osteoporosis (yr)		5.80 ± 1.80	5.47 ± 1.83	1.093	.276
First fracture	Yes	60 (84.51%)	60 (81.08%)	0.298	.585
	No	11 (15.49%)	14 (18.92%)	–	–

PMT = protection motivation theory.

### 3.2. Attenuation in postoperative inflammation in the PMT group

**Figure 1.**
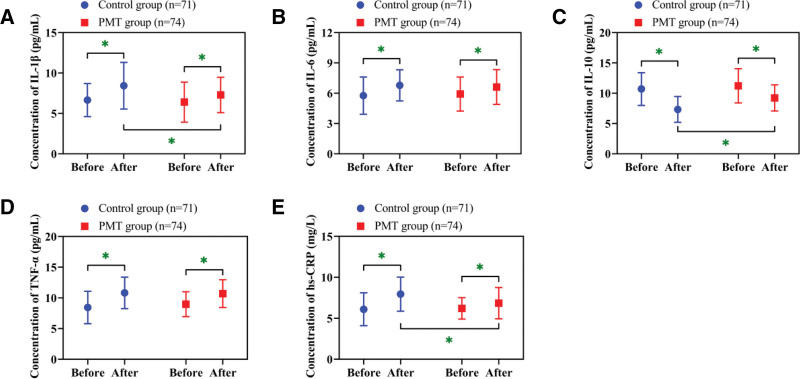
Changes and comparison of inflammatory factors. (A) Changes and comparison of IL-1β concentrations. (B) Changes and comparison of IL-6 concentrations. (C) Changes and comparison of IL-10 concentrations. (D) Changes and comparison of TNF-α concentrations. (E) Changes and comparison of hs-CRP concentrations. * *P* < .05. hs-CRP = high-sensitivity C-reactive protein, IL = interleukin, TNF-α = tumor necrosis factor-α.

Changes in inflammatory markers were assessed pre- and posttreatment. An increase in IL-1β, IL-6, TNF-α, and hs-CRP, coupled with a decrease in IL-10, was observed in all subjects after surgery (*P* < .05). While preoperative cytokine profiles and postoperative IL-6 and TNF-α did not differ significantly between groups (*P* > .05), the PMT cohort demonstrated significantly reduced IL-1β and hs-CRP, as well as increased IL-10 levels relative to controls following treatment (*P* < .05; Fig. 1).

### 3.3. Reduction in postoperative pain in the PMT group

**Figure 2.**
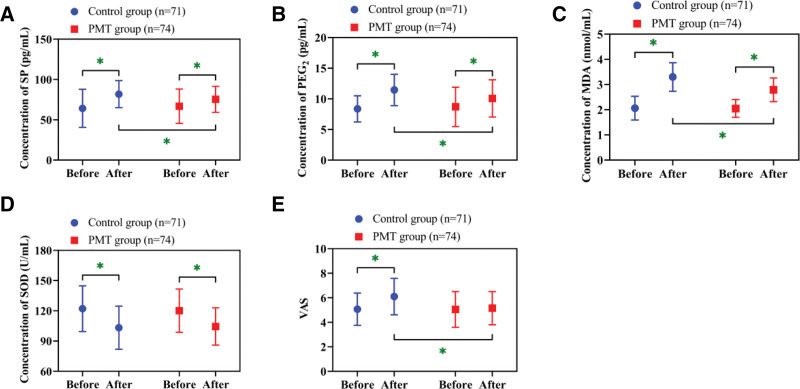
Changes and comparison of pain mediators. (A) Changes and comparison of SP concentrations. (B) Changes and comparison of PGE_2_ concentrations. (C) Changes and comparison of MDA concentrations. (D) Changes and comparison of SOD concentrations. (E) Changes and comparison of VAS. * *P* < .05. MDA = malondialdehyde, PGE_2_ = prostaglandin E, SOD = superoxide dismutase, SP = substance P, VAS = visual analogue scale.

Analysis of pain mediators revealed elevated postoperative concentrations of SP, PGE_2_, and MDA, along with reduced SOD activity in both groups (*P* < .05). While baseline levels of these markers were comparable between groups (*P* > .05), the PMT group demonstrated significantly lower postoperative SP, PGE_2_, and MDA relative to controls (*P* < .05). Similarly, there was no significant difference in preoperative VAS between the 2 groups (*P* > .05). After operation, the VAS of the PMT group did not change significantly and was lower than that of the control group (*P* < .05), while the VAS of the control group after operation was higher than that before operation (*P* < .05; Fig. 2).

### 3.4. Amelioration in postoperative hypercoagulability in the PMT group

**Figure 3.**
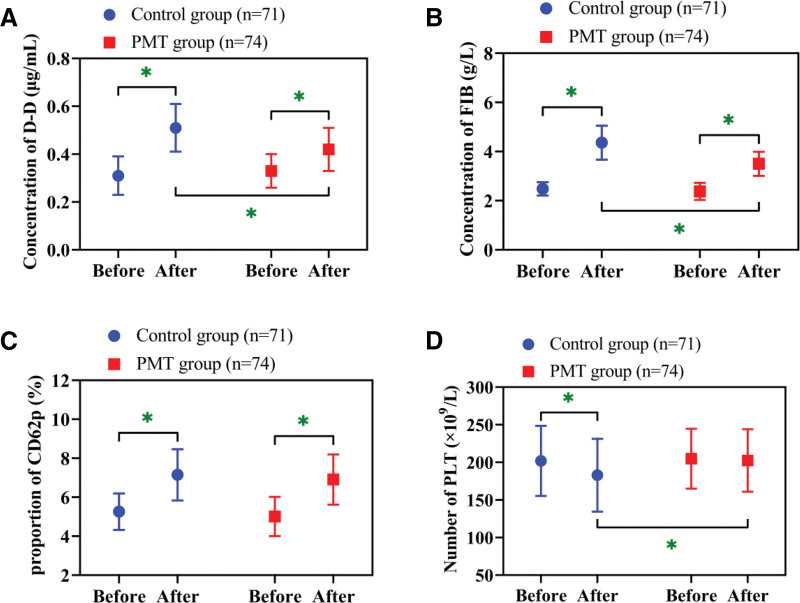
Changes and comparison of coagulation function. (A) Changes and comparison of D-D concentrations. (B) Changes and comparison of FIB concentrations. (C) Changes and comparison of CD62p proportions. (D) Changes and comparison of PLT numbers. * *P* < .05. CD62p = P-selectin, D-D = D-dimer, FIB = fibrinogen, PLT = platelet.

Hemodynamic analyses revealed significant postoperative increases in D-D, FIB, and P-selectin compared with preoperative levels (*P* < .05). PLT declined significantly in the control group (*P* < .05), but remained stable in the PMT group (*P* > .05). Intergroup comparisons showed that the PMT group had lower postoperative D-D and FIB and higher PLT levels than the control group (*P* < .05; Fig. 3).

### 3.5. Restoration of bone metabolism markers in the PMT group

**Figure 4.**
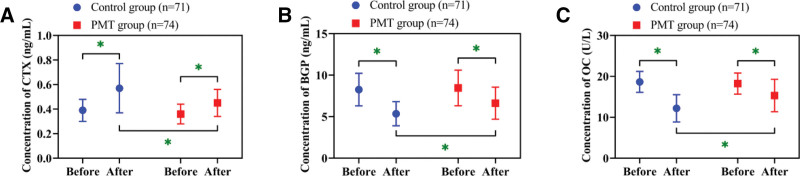
Changes and comparison of bone metabolism markers. (A) Changes and comparison of CTX concentrations. (B) Changes and comparison of BGP concentrations. (C) Changes and comparison of OC concentrations. * *P* < .05. BGP = bone Gla protein, CTX = C-terminal telopeptide of type I collagen, OC = osteocalcin.

Evaluation of bone turnover markers indicated a postoperative rise in CTX and declines in BGP and OC in both groups (*P* < .05). No between-group differences were detected in CTX, BGP, or OC at baseline (*P* > .05). After treatment, however, the PMT group showed lower CTX and higher BGP and OC levels compared with the control group (*P* < .05; Fig. 4).

### 3.6. The perioperative safety of PMT group is higher

According to statistics, DVT, dizziness, headache, and other events occurred in the PMT group and the control group during the perioperative period. Compared with the control group, the incidence of DVT in the PMT group was lower (*P* < .05; Table 2).

**Table 2:** Comparison of adverse effects.

Projects		DVT	Dizziness/headache	Postoperative infection	Abdominal pain/diarrhea	Nerve/spinal cord injury	Bone cement leakage
Control group	71	9 (12.68)	8 (11.27)	1 (1.41)	4 (5.63)	3 (4.23)	2 (2.82)
PMT group	74	2 (2.70)	6 (8.11)	0 (0.00)	3 (4.05)	1 (1.35)	2 (2.82)
χ^2^	–	–	0.415	–	–	–	–
*P*	–	.029	.520	.490	.715	.360	1.000

DVT = deep venous thrombosis, PMT = protection motivation theory.

### 3.7. The quality of postoperative rehabilitation was better in the PMT group

Finally, the rehabilitation status of the 2 groups was observed, and the JOA and BI of the 2 groups were higher than those before operation (*P* < .05). The postoperative JOA and BI in the PMT group were 22.01 ± 3.18 and 75 (65, 75), which were higher than those in the control group (*P* < .05; Table 3).

**Table 3:** Comparison of BI and JOA scores.

Projects		Control group (n = 71)	PMT group (n = 74)	Statistics, *t* or *U*	*P*
JOA	Before	16.90 ± 3.17	16.47 ± 3.55	0.765	.446
	After	20.37 ± 2.92	22.01 ± 3.18	3.245	.002
BI	Before	60 (55, 65)	60 (55, 65)	2415.069	.392
	After	70 (65, 75)	75 (65, 75)	2004.351	.004

BI = Barthel index, JOA = Japanese Orthopaedic Association.

## 4. Discussion

This study establishes the efficacy of the PMT management model in the multifaceted regulation of pathophysiological markers during the early postoperative phase following OVF surgery. It offers a novel perspective for postoperative management, with great clinical implications for optimizing the postoperative care pathways of OVF and augmenting rehabilitation efficiency.

The study showed lower IL-1β and hs-CRP (pro-inflammatorymarkers) as well as higher IL-10 (an anti-inflammatory cytokine) in the PMT group versus the control group following surgery. These outcomes are consistent with the concept that PMT reduces stress through cognitive restructuring.^[13]^ Through threat appraisal, patients become aware of the risks associated with excessive inflammation, which motivates them to participate proactively in early mobilization. Such physical activity suppresses pro-inflammatory cytokine release through muscular contractions.^[14]^ In addition, PMT’s coping appraisal reinforces self-efficacy via personalized daily activity goals, further alleviating inflammation. Similarly, research by Morowatisharifabad et al^[8]^ on diabetes mellitus patients indicated that PMT-based strategies can lower IL-6, underscoring its regulatory impact on inflammation.

Furthermore, the PMT group demonstrated more significant postoperative reductions in SP, PGE_2_, and MDA compared with the control group, indicating PMT’s superiority in mitigating postoperative pain. The release of pain mediators correlates closely with inflammatory responses: pro-inflammatory mediators like IL-6 are known to stimulate nerve endings to release SP,^[15]^ while PGE_2_ amplifies nociceptive transmission via the cyclooxygenase pathway.^[16]^ PMT contributes to the suppression of these pain-related molecules by mitigating inflammatory responses. In addition, motivational interventions may modulate autonomic nervous system activity and decrease oxidative stress, thereby reducing pain perception. In a study by Owen et al,^[17]^ individuals receiving PMT showed decreased PGE_2_ levels through improved psychological adaptability, aligning with the current findings. Similarly, the results of VAS between the 2 groups were also consistent with the trend of pain mediators, which again supported the protective effect of PMT on postoperative pain.

Postoperative assessments further revealed lower D-D and FIB, along with higher PLT levels in the PMT group relative to controls. Hence, PMT is also effective in alleviating the postoperative hypercoagulable state of patients. Postoperative hypercoagulability is a major cause of DVT.^[18]^ Under PMT-based interventions, patients are encouraged to engage in early activities through behavioral reinforcement strategies like setting a daily standing time goal, thus enhancing blood flow velocity. Meanwhile, the threat appraisal component of PMT enhances patients’ willingness to comply with anticoagulation therapy (e.g., low-molecular-weight heparin) by clarifying the potential risks of hypercoagulability, further reducing thrombotic risk. We speculate that this is one of the reasons why the incidence of postoperative DVT was lower in the PMT group than in the control group. Not only that, previous studies have also shown that systematic PMT can effectively reduce the incidence of postoperative adverse reactions in patients undergoing knee replacement,^[19]^ emphasizing the positive role of PMT in surgical safety. However, this paper only found a difference in the incidence of DVT between the 2 groups, which may be speculated to be due to the small number of cases. We will expand the sample size for further validation analysis.

We observed lower postoperative CTX (a marker of bone resorption) in the PMT group compared with controls, together with higher BGP (a marker of osteogenesis) and OC (a marker of mineralization). This suggests that PMT positively influences the correction of bone metabolic imbalance – a condition largely driven by overactive osteoclasts and impaired osteoblast performance.^[20]^ By improving patients’ awareness of the adverse effects of bone metabolic imbalance, PMT encourages higher adherence to anti-osteoporosis medication, thereby helping to suppress osteoclast activity. Furthermore, early activities promote the proliferation of osteoblasts through mechanical stimulation,^[21]^ forming a positive bone metabolism cycle. This is consistent with the theory of “positive correlation between exercise and bone metabolism” proposed by Zhang et al.^[22]^

Finally, in the investigation of rehabilitation status, although JOA and BI increased in both groups at 1 month after surgery, the increase in the PMT group was greater than that in the control group, emphasizing the positive role of PMT in improving the prognosis of OVF patients. Studies have shown that PMT promotes early activity of patients through “behavioral benefit reinforcement” and, combined with the use of anti-osteoporosis drugs, accelerates the recovery of muscle strength and improves spinal stability.^[17]^ At the same time, combined with the positive effects of PMT on pain relief and anti-inflammation at the physiological level, it lays a reliable foundation for the prognosis and rehabilitation of patients to the greatest extent. Estebsari et al also obtained similar results in this paper when exploring the postoperative application of PMT in breast cancer patients.^[6]^

According to the study’s findings, the research team proposed the incorporation of the PMT management model into the standard postoperative care pathway for OVF, aiming to enhance recovery outcomes for such patients. However, this study is subject to certain limitations that warrant further investigation. For example, the observation period only covers 5 days after surgery, so it is impossible to track PMT’s impact on long-term complications. In addition, the limited sample size prevented subgroup analyses based on surgical techniques, making it difficult to assess whether the intervention effect varied across different procedures. Moreover, the extensive use of enzyme-linked immunosorbent assays in this study may have introduced false positives resulting from antibody cross-reactivity. Subsequent studies should extend the follow-up period, increase the sample size, and incorporate multiple control groups to confirm PMT’s long-term benefits and specificity. It should be noted that early postoperative biomarker fluctuations may partially reflect surgical stress responses rather than solely the effects of PMT-based management. Although intergroup comparisons suggest a modulatory role of PMT, longer follow-up periods are necessary to clarify its independent physiological impact.

## 5. Conclusion

Early application of PMT after OVF surgery can inhibit inflammation, decrease pain mediator secretion, ease blood hypercoagulability, and correct bone metabolism irregularities, so as to lay a more reliable foundation for the prognosis and rehabilitation of patients. This finding provides a new nonpharmacological intervention strategy for breaking the pathological vicious cycle after OVF surgery, with considerable clinical translation potential. Nonetheless, further validation of PMT’s durable efficacy and a deeper investigation into how it synergizes with novel therapies remain essential.

## Author contributions

**Conceptualization:** Xiaorong Guo, Xuan Wei, Xiaojuan Zhang, Jing Liu.

**Data curation:** Xiaorong Guo, Xuan Wei, Xiaojuan Zhang, Jing Liu.

**Formal analysis:** Xiaorong Guo, Xuan Wei, Xiaojuan Zhang, Jing Liu.

**Funding acquisition:** Xiaorong Guo, Xuan Wei, Jing Liu.

**Investigation:** Xiaorong Guo, Jing Liu.

**Writing – original draft:** Xiaorong Guo, Xuan Wei, Xiaojuan Zhang, Jing Liu.

**Writing – review & editing:** Xiaorong Guo, Xuan Wei, Xiaojuan Zhang, Jing Liu.
